# Conference Calendar

**DOI:** 10.1002/cfg.122

**Published:** 2001-12


					Conference Calendar
November 2001-February 2002
Animal and plant models
CSHL - Physiological Genomics and Rat
Models - Dec 6-9 2001
http://nucleus.cshl.org/meetings/2001Rodent.htm
Plant, Animal and Microbe Genomes X - Jan
12-16 2002
http://www.intl-pag.org/pag/
Bacteria, yeasts and fungi
9th
International Conference on Microbial
Genomes - Oct 28-Nov 1 2001
http://www.esd.ornl.gov/microbial_genomes/
index.html
6th ASM Conference on Candida and
Candidaisis - Jan 13-17 2002
http://www.asmusa.org/mtgsrc/6thCandidag.htm
2nd
ASM and TIGR Conference on Microbial
Genomes - Feb 10-13 2002
http://www.asmusa.org/mtgsrc/MG2g.htm
Bioinformatics
ESF - Workshop on Information Extraction in
Molecular Biology - Nov 11-14 2001
http://www.functionalgenomics.org.uk/sections/activi-
tites/workshops.htm
IQPC - BioForums: Integrating Bioinformatics
and Cheminformatics - Nov 14-15 2001
http://www.iqpc.com/cgi-bin/templates/genevent.html?
topic=8&event=1904
TIGR - Computational Genomics - Nov
29-Dec 1 2001
http://www.tigr.org/cet/gss/cg/index.shtml
12th Workshop on Genome Informatics (GIW
2001) - Dec 18-19 2001
http://giw.ims.u-tokyo.ac.jp/giw2001/index.html
Pacific Symposium on Biocomputing (PSB)
2002 - Jan 3-7 2002
http://psb.stanford.edu/
CHI - High Performance Computing - Jan
14-15 2002
http://www.healthtech.com/2002/hpc/index.htm
CHI - Integrated Bioinformatics - Jan 16-18
2002
http://www.healthtech.com/2002/bne/index.htm
The International Conference on
Bioinformatics: North-South Network - Feb
6-8 2002
http://incob.biotec.or.th/
Evolution/comparative genomics
Annual Meeting of the Society for Integrative
and Comparative Biology - Jan 6-10 2002
http://www.sicb.org/meetings/2002/index.php3
Genomics
IBC - Biomics (Genomics and Proteomics
Conference)- Nov 13-16 2001
http://www.ibc-biomics.com/
Comparative and Functional Genomics
Comp Funct Genom 2001; 2: 398-400.
DOI: 10.1002/cfg.122
Published online: 14 November 2001
Copyright # 2001 John Wiley & Sons, Ltd.
Reproductive Genomics - Dec 5-6 2001
http://www.monashinstitute.org/news/
PHIMR,IRDSymposium/index.html
1st
Annual TIGR Functional Genomics Meeting
- Jan 17-20 2002
http://www.tigr.org/cet/gss/fg/
IQPC - The Next Step in Functional
Genomics and Proteomics - Jan 29-30 2002
http://www.iqpc.com/cgi-bin/templates/genevent.html?
topic=8&event=2021
Advances in Genome Biology and Technology
- Feb 6-9 2002
http://www.agbt.org
Human
CHI - 2nd Annual Human Proteome Project -
Jan 9-11 2002
http://www.chi-peptalk.com/
CHI - Genome Tri-Conference - Feb 23-Mar
1 2002
http://www.genometriconference.com/index2.htm
Integrative and systems biology
2nd International Conference on Systems
Biology (ICSB2001) - Nov 5-7 2001
http://www.icsb2001.org/
CHI - Integrated Bioinformatics - Jan 16-18
2002
http://www.healthtech.com/2002/bne/index.htm
Metabolomics
CHI - Metabolic Profiling - Pathways to
Discovery - Dec 3-4 2001
http://www.healthtech.com/2001/mbp/index.htm
Pharmacogenomics
IBC - Realizing Genomic Medicine - Dec 3-4
2001
http://www.genomic-med.com/
SRI - Pharmacogenomics and SNPs - Jan
28-29 2002
http://www.srinstitute.com/part_iter_site_page.cfm?
iteration_id=274
Proteomics
CHI - Protein Informatics - Nov 12-13 2001
http://www.healthtech.com/2001/pnf/index.htm
CHI - Pep Talk (including 2nd
Annual Human
Proteome Project) - Jan 7-11 2002
http://www.chi-peptalk.com/
Structural genomics
Keystone - Structural Genomics: From gene
sequence to function - Jan 5-11 2002
http://www.symposia.com/Meetings/ViewMeetings.
cfm?MeetingID=598
Keystone - Frontiers of Structural Biology -
Jan 5-11 2002
http://www.symposia.com/Meetings/ViewMeetings.
cfm?MeetingID=599
Transcriptomics
CHI - Microarray Data Analysis - Nov 12-13
2001
http://www.healthtech.com/2001/mda/index.htm
CHI - Lab-on-a-Chip and Microarrays - Jan
14-16 2002
http://www.healthtech.com/2002/mfe/index.htm
4th Microarray Gene Expression DataBase -
Feb 2002
http://www.mged.org/
Conference Calendar 399
Copyright # 2001 John Wiley & Sons, Ltd. Comp Funct Genom 2001; 2: 398-400.
Key
ASM- American Society for Microbiology [http://
www.asmusa.org]
CHI- Cambridge Healthtech Institute [http://www.
healthtech.com]
CSHL- Cold Spring Harbor Laboratory [http://
www.cshl.org]
ESF- European Science Foundation [http://www.esf.
org]
IBC- IBC conferences [http://www.ibcusa.com]
IQPC- International Quality and Productivity Center
[http://www.iqpc.com]
SRI- Strategic Research Institute [http://www.srinstitute.
com]
TIGR- The Institute for Genome Research [http://
www.tigr.org]
The Conference Calendar is a listing of forthcoming conferences of interest to our readership. We provide
the title, dates and web address of each conference, to enable readers to find out more information.
Inclusion does not imply endorsement by the Journal. If you know of a relevant conference, please contact
the Managing Editor.
www.wiley.co.uk/genomics
The Genomics website at Wiley is a DYNAMIC resource for the genomics community, offering
FREE special feature articles and new information EACH MONTH.
Find out more about our new journals Comparative and Functional Genomics, and Proteomics.
Visit the Library for hot books in Genomics, Bioinformatics, Molecular Genetics and more.
Click on Journals for information on all our up-to-the minute journals, including: Genesis,
Bioessays, Journal of Mass Spectrometry, Gene Function and Disease, Human Mutation, Genes,
Chromosomes and Cancer and the Journal of Gene Medicine.
Let the Genomics website at Wiley be your guide to genomics-related web sites, manufacturers and
suppliers, and a calendar of conferences.
The
Website at Wiley
2380
400 Conference Calendar
Copyright # 2001 John Wiley & Sons, Ltd. Comp Funct Genom 2001; 2: 398-400.

				
